# Diagnosis of Cushing’s syndrome with generalized linear model and development of mobile application

**DOI:** 10.1097/MD.0000000000042910

**Published:** 2025-06-20

**Authors:** Mustafa Aydemir, Mustafa Çakir, Okan Oral, Mesut Yilmaz

**Affiliations:** a Department of Internal Medicine, Division of Endocrinology and Metabolism, Akdeniz University School of Medicine Antalya, Antalya, Turkey; b Iskenderun Technical University, Iskenderun Vocational School of Higher Education, Iskenderun, Hatay, Turkey; c Akdeniz University, Faculty of Engineering, Mechatronics Engineering Antalya, Turkey; d Department of Aquaculture, Faculty of Aquaculture, Akdeniz University, Antalya, Turkey.

**Keywords:** Cushing Diagnosis Mobile App, Cushing disease, Cushing syndrome, ectopic Cushing syndrome, machine learning

## Abstract

**Background:**

Cushing syndrome (CS) is a rare endocrine disorder characterized by excessive secretion of glucocorticoids, leading to a variety of clinical manifestations, comorbidities, and increased mortality despite treatment. Despite advances in imaging modalities and biochemical testing, the diagnosis and management of CS remains challenging. Several tests are used to confirm the diagnosis of CS, including urinary free cortisol measurements, dexamethasone suppression tests (1 mg, 2 mg, and 8 mg), and nocturnal salivary cortisol measurements. However, each of these tests has some limitations, making the diagnosis of CS.

**Methods:**

In this paper, we explore the potential of state-of-the-art machine learning algorithms as a clinical decision support system for analyzing and classifying CS. Our aim is to use advanced machine learning methods to analyze the accuracy rates of diagnostic tests and identify the most sensitive tests for diagnosing CS.

**Results:**

In this study, we performed binary classification based on data from 278 patients with CS (CS+) and 220 healthy patients (CS-). We developed a linear mathematical model with high predictive ability, achieving a classification accuracy of 97.03% and a Kappa value of 94.05%. The correlation graph shows that CS has strong positive relationships with 2 mg (78.8%), 1 mg (76.9%), and mc (72.1%), and moderate positive correlations with 8 mg (45%) and saliva (45.4%). In contrast, gender has almost no correlation with CS, so it was removed from the dataset. As a result, the model achieves an overall classification accuracy of 97.03%. Finally, we converted the linear model into a mobile application for use by specialist doctors in the field of endocrinology.

**Conclusion:**

Traditional diagnostic methods can be time-consuming and require specialized medical expertise. Recently, advances in machine learning and mobile technology have opened new avenues for improving diagnostic accuracy and accessibility. This study explores the integration of machine learning algorithms into a mobile application designed to assist healthcare professionals and patients in the diagnosis of CS.

## 1. Introduction

Cushing syndrome (CS) is a potentially fatal disease caused by abnormally high levels of the cortisol hormone, first described by Harvey Cushing in 1912.^[1–6]^ The estimated global incidence of CS ranges from 1.8 to 4.5 cases per million people per year, with a prevalence of 57 to 79 cases per million people per year when considering all causes. Registry data indicate a female-to-male ratio of 4:1 and a mean age at diagnosis of 44 years (±14).^[4,7]^

Exogenous CS can occur due to long-term intake of glucocorticoid-steroid hormones, which are prescribed for conditions such as asthma, rheumatoid arthritis, lupus, and other inflammatory diseases.^[8]^ These hormones, which are chemically similar to natural cortisol, have potent anti-inflammatory properties. They are also utilized post-organ transplantation to suppress the immune system and prevent rejection. Endogenous causes involve the body’s excessive production of cortisol. In pituitary-induced Cushing (PC), excessive secretion of ACTH stimulates the adrenal glands to produce cortisol. PC is the most common cause of Cushing syndrome and is also referred to as Cushing disease. Adrenal Cushing (AC) can be due to adrenal tumors or adrenal hyperplasia, a genetic disorder leading to cortisol overproduction. Ectopic CS (EC) occurs when tumors located outside the pituitary gland, such as those in the pancreas, lungs or thyroid produce ACTH.^[8]^ The majority (70–80%) of endogenous CS cases are classified into ACTH-dependent and ACTH-independent causes. Cushing disease, the most common form of ACTH-dependent CS (80–90%), is also known as corticotroph pituitary adenoma or corticotropinoma.^[9]^ Non-pituitary ACTH-secreting tumors (ectopic ACTH secretion) represent 10% to 20% of ACTH-dependent cases. Lung tumors, particularly neuroendocrine tumors, account for a significant portion of ectopic ACTH production cases.^[4,8,9]^

ACTH-independent CS is primarily caused by unilateral adrenal adenomas (70%) and adrenal carcinomas (20–30%).^[10,11]^ Rare causes include bilateral macronodular adrenocortical disease, bilateral micronodular adrenal hyperplasia (isolated or part of the Carney complex), McCune–Albright syndrome, and bilateral adrenal adenomas or carcinomas.^[12–14]^

Early diagnosis plays a crucial role in reducing mortality and improving the prognosis of CS. However, diagnosing CS can be challenging due to symptoms developing gradually and overlapping with features of metabolic syndrome, such as high blood pressure, elevated blood sugar, excess abdominal fat, and abnormal cholesterol, and triglyceride levels.^[15]^ Due to the nonspecific nature of these symptoms, diagnosing CS can be challenging. Laboratory investigations for clinically suspected CS patients are divided into 2 stages. Stage-1 tests serve as diagnostic screening tests to prove the presence of hypercortisolism, while Stage-2 tests are follow-up tests to evaluate the cause of hypercortisolism.^[16–18]^

The most commonly used procedures to evaluate and exclude or confirm CS include the urinary cortisol test, which measures cortisol levels in a 24-hour urine sample, the salivary cortisol test, which measures cortisol levels in saliva and the low-dose dexamethasone supression test, which measures cortisol levels in blood after ingestion of dexamethasone. Despite their predictive value, test results can still be inconclusive. Typically, 3 tests are recommended for screening: late-night salivary cortisol, urinary free cortisol, and an overnight 1 mg dexamethasone suppression test. Most patients require at least 2 positive test results to confirm hypercortisolism, with each test having its own characteristics and limitations. Inconclusive results may occur in patients in the early stages of the disease or with periodic forms of CS, sometimes necessitating prolonged follow-up or hospitalization.^[18–20]^

Diagnosing and determining the cause of CS requires careful interpretation of signs, symptoms, biochemical test results, and medical imaging findings by experienced physicians. Traditional diagnostic approaches rely on statistical methods and different cutoff points for sensitivity and specificity, leading to varying evaluations of test results.^[21]^ ML offers a promising solution to overcome these challenges by providing a generalizable approach to evaluate medical test results, predict CS diagnosis, and aid in prognosis. This paper explores state-of-the-art ML algorithms and demonstrates their utility as a clinical decision support system for the diagnosis and prognosis of CS. Clinical performance evaluation is conducted by comparing model’s predictions with expert physicians judgments.^[21,22]^

The integration of artificial intelligence (AI) models in the diagnosis of medical conditions has gained substantial momentum in recent years. A notable example is the widespread application of machine learning algorithms during the COVID-19 pandemic, where these tools played a pivotal role in supporting disease detection and diagnosis.^[23]^ Beyond infectious disease contexts, AI has shown considerable promise in neuropsychiatric domains. For instance, the diagnosis of conditions such as Schizophrenia and Attention Deficit Hyperactivity Disorder has been investigated using resting-state functional magnetic resonance imaging data. In these applications, advanced AI techniques like two-dimensional Convolutional Autoencoders have been employed to perform efficient and informative feature extraction.^[24]^ To further improve diagnostic precision and model transparency, hybrid approaches have been proposed. One such example combines Interval Type-2 Fuzzy Regression with Gray Wolf Optimization, yielding enhanced classification performance and better interpretability. These developments underscore the increasing influence of AI in optimizing diagnostic workflows across both somatic and mental health care settings.^[24]^

Moreover, in recent years, nonlinear features (such as fractal dimension and entropy) have emerged as critical elements in biomedical data analysis, significantly contributing to the improvement of diagnostic accuracy.^[25]^ These features help capture the complex, irregular patterns commonly observed in physiological signals and medical imaging, making them especially valuable for detecting subtle pathological changes.^[25,26]^

Fuzzy logic-based models have also become increasingly prominent in clinical decision support systems, primarily due to their inherent ability to manage vagueness and uncertainty within medical data. Techniques such as fuzzy regression (particularly hierarchical fuzzy systems and Takagi–Sugeno–Kang (TSK) models) offer a flexible framework for modeling the intricate and often nonlinear interactions among clinical variables.^[27]^ TSK fuzzy systems, in particular, have demonstrated strong performance in handling nonlinear dynamics within healthcare datasets, frequently surpassing traditional linear models in both accuracy and adaptability.^[28]^ Compared to conventional linear models, these approaches frequently excel in both accuracy and adaptability. The use of fuzzy concepts in handling uncertainties in medical data can lead to more accurate and reliable outcomes.^[29–31]^

Despite these advantages, the implementation of sophisticated fuzzy and AI-based models typically requires significant computational resources and access to specialized development platforms.^[29–31]^ In response to this challenge, our study focused on developing a more practical and resource-efficient solution using Generalized Linear Models (GLM), which enabled seamless integration with the MIT App Inventor platform. This approach provides a lightweight yet effective alternative, ensuring broader accessibility and applicability in real-world clinical settings.

### 1.1. Related study

ML approaches have been applied to various problems related to CS.^[32]^ Previous studies have explored automated interpretation of urinary steroid profiles to classify normal and abnormal profiles associated with various metabolic conditions, including CS.^[33]^ Classification of CS using gene expression data of tumor tissues has been demonstrated.^[34]^ Furhermore, ML has been used to identify predictors of early and long-term outcomes after surgery in patients treated for Cushing disease.^[35]^ Another study focused on identifying facial anomalies associated with endocrinal disorders, including CS, using ML to facilitate diagnosis and follow-up.^[36]^

In this study, our aim was to analyze the tests used to diagnose CS using advanced ML methods, determine their accuracy rates, and identify which tests exhibit higher sensitivity in diagnosing CS. This is the first study to present comprehensive research on the application of the ML approach in this context. An AI model was developed based on data from various diagnostic tests, including the 1 mg dexamethasone suppression test (DST), 2 mg DST, 8 mg DST, basal cortisol level, midnight cortisol, night salivary free cortisol, 24-hour urine free cortisol and basal ACTH levels. The obtained mathematical classification model was converted into a mobile application for use by endocrinology specialists. To the best of our knowledge, there is no previous study that has transformed an ML-derived model into a mobile application, making this research original.

## 2. Materials and methods

### 2.1. Study design

Retrospective medical records of 498 patients (319 women and 179 men, with a mean age of 52.02 ± 13.33 years) were used. These patients were admitted to the Akdeniz University Faculty of Medicine Endocrinology outpatient clinic between 2014 and 2023 due to symptoms of Cushing syndrome or incidental adrenal adenomas. Diagnostic test results, including basal cortisol, basal ACTH, 1 mg DST cortisol, 2 mg DST cortisol, 8 mg DST cortisol, midnight cortisol, 24-hour urine cortisol, and adrenal and pituitary imaging tests, were analyzed. The Akdeniz University Faculty of Medicine Ethics Committee approved this research study (TBAEK-250 25.04.2024).

### 2.2. Dataset profile

The CS dataset comprises 498 samples and 11 features (predictor variables). The target variable represents 4 subtype classes: AC, PC, subclinical Cushing sydrome (SC), and EC for patients with CS, and nonfunctional adrenal adenoma (NF) for patients without CS. Statistical characteristics (mean, standard deviation, proportion, and numbers) of dataset characteristics by diagnosis type are provided in Table 1. The dataset contains missing values because some medical tests were not performed based on doctors’ orders, depending on symptoms and accompanying test results. Some of the data distributions were skewed (asymmetric around the mean of the distribution). The dataset also suffers from moderate class imbalance, as the NF class is overrepresented compared to the other classes.

**Table 1:** Characteristics of patients at time of diagnosis data set.

Feature	Description	NF (n:220)		AC (n:114)	PC (n:89)	EC (n:16)	SC (n:59)
		MVR%	Mean	Mean	Mean	Mean	Mean
Age	Subject age (years)	0	47.89 ± 15.62	60.24 ± 13.27	51.48 ± 14.40	46.13 ± 16.62	58.51 ± 10.52
Gender	Subject gender (female/male count)	0	156/64	86/28	66/23	11/5	47/12
Saliva	Night salivary cortisol	0	0.19 ± 0.10	0.60 ± 1.19	0.47 ± 0.38	3.88 ± 8.12	0.39 ± 0.20
bc	Basal cortisol (µg/dL)	29	16.15 ± 5.10	18.71 ± 7.52	22.79 ± 9.62	36.64 ± 15.60	17.29 ± 4.88
Batch	Basal ACTH (pg/mL)	35	21.32 ± 14.59	5.57 ± 3.19	77.38 ± 149.8	224.63 ± 265.72	4.82 ± 3.70
1 mg DSTc	Cortisol after 1 mg DST (µg/dL)	5	1.35 ± 1.17	7.34 ± 7.88	8.38 ± 7.43	18.57 ± 19.93	4.54 ± 3.75
2 mg DSTc	cortisol after 2 mg DST (µg/dL)	23	1.01 ± 0.42	7.27 ± 8.35	8.35 ± 7.3	19.28 ± 20.85	4.44 ± 4.00
8 mg DSTc	8 mg DST	68	1.27 ± 0.21	0.90 ± 0	6.96 ± 3.22	26.50 ± 14.96	4.07 ± 2.46
mc	Midnight cortisol (µg/dL)	68	3.46 ± 1.64	11.98 ± 7.75	14.98 ± 9.49	27.55 ± 22.01	10.20 ± 6.41
ufc	Urinary free cortisol (µg/24 h)	29	63.71 ± 42.52	228.72 ± 275.0	331.69 ± 263.47	1111.79 ± 1286.71	120.71 ± 71.19
adrMass	Mass in adrenal imaging (yes/no)	0	220/0	114/0	0/89	16/0	59/3
pitMass	Mass in pituitary imaging (yes/no)	0	0/220	0/114	89/0	16/0	3/59

AC = adrenal Cushing, DST = dexamethasone suppression test, EC = ectopic Cushing, MVR = missing value imputation, NF = nonfunctional adrenal adenoma for patients without CS, PC = pituiter Cushing, SC = subclinical Cushing sydrome.

### 2.3. Preprocessing

#### 2.3.1. Missing value imputation

Physicians may omit certain tests if previous tests provide sufficient diagnostic information. For instance, the 8 mg DST test is typically only conducted following screening tests like the 1 mg DST to ascertain the type of CS. If a person is suspected of having a nonfunctioning adrenal adenoma, the 8 mg DST is often omitted. Depending on symptom severity and other indicators, the physician may opt to skip the low-dose DST test and proceed directly to the 8 mg DST test. Therefore, we opted against skipping features with missing values and instead employed imputation.

Dealing with missing data poses a significant statistical challenge, and there is no one-size-fits-all imputation method that works best in all scenarios.^[37]^ The simplest approach is to discard samples with missing values. However, in this study, discarding samples with missing feature values was not considered due to the prevalence of missing values in the original dataset. Such exclusions would lead to substantial information loss and a significantly reduced dataset size. To address missing values for a given feature, we replaced them with the median of all known values for that feature, calculated separately for both the training and validation datasets. This approach avoids information leakage and maintains the dataset’s independence throughout the training process.

Before modeling the dataset with the ML algorithm, it is essential to examine the dataset’s general structure and perform necessary operations. Statistical methods are employed during the data overview phase to gain insights into the general distribution of features in the dataset. At this stage, if any missing data entries are identified, the relevant observations are retained in the dataset. Following the data cleaning process, the dataset is finalized by conducting operations such as logarithmic transformation and outlier detection, if necessary.

#### 2.3.2. Skewed feature distributions problem

In general, skewed feature distributions in the dataset reduce the model’s ability to identify more common cases, as it focuses more on dealing with rarer cases that take extreme values. Some ML algorithms (e.g., the LDA classifier) assume normality for the underlying populations, and their performance can be negatively impacted by violations of this assumption.^[38]^

Classifiers without distributional assumptions are expected to perform well with various distributions, as long as the class distributions are reasonably distinct.^[39]^ In a similar study, logarithmic transformation was employed to mitigate skewed distributions, resulting in improved performance. We applied a logarithmic transformation (log base 10) to reduce skewness of feature distributions and reported the level of skewness using the Fisher–Pearson skewness coefficient.^[40,41]^

After preprocessing, correlation analysis was applied to measure the relationships between features in the dataset, revealing how the dependent variable correlates with the independent variables.

### 2.4. Machine learning classification and evaluation method

GLM is one of commonly used ML algorithms for classification problems. It differs from other ML algorithms, called blackbox algorithms, in that it creates a linear model and provides the weight coefficients of the features as a result, which can be easily interpreted. The β coefficients of the features in the mathematical model are determined. After determining the coefficients, a logit transformation is performed.^[42]^ To calculate probabilities using the obtained logit values, a transformation is made as described in formula (1):

$$\begin{array}{l} logit\left(\;p\right)=\beta_{0}+\;\beta_{1}.age+\beta_{2}.saliva+\beta_{3}.bc+\beta_{4}.bacth\;\\ \;\;\;\;\;\;\;\;\;\;\;\;\;\;\;\;\;\;+\beta_{5}.mg1+\beta_{6}.mg2+\beta_{7}.mg8+\beta_{9}.mc+\beta_{10}.ufc\; \end{array}$$  (1)

Where Y is the dependent variable, that is, CS, and X is the independent variables, that is, {age, saliva, basal cortisol (bc), basal acth (bacth), 1 mg DST (mg1), 2 mg DST (mg2), 8 mg DST (mg8), midnigt cortisole (mc), urinary free cortisol (ufc)}, the probability of a patient being CS + is calculated as in formula (2).^[43]^ If the calculated probability value is >0.5, then the patient is considered CS+, and if it is <0.5, then the patient is considered

$$\begin{array}{l} CS-.\;\;P\left(Y=1X\right)=\;\frac{1}{1+\;e^{-logit\left(\;p\right)}} \end{array}$$ (2)

The success of a classification model needs to be tested with available data. Among the most frequently utilized metrics in the field of classification are Accuracy, Kappa, Sensitivity, Specificity, Positive Prediction Value, Negative Prediction Value, Prevalence, Detection Rate, Detection Prevalence, and Balanced Accuracy.^[44]^ These metrics provide a comprehensive understanding of the model’s performance. A Confusion Matrix (Table 2) is used to mathematically express these evaluation metrics. It is a table that illustrates how the model combines actual and predicted classes. The fundamental terms in this table are defined as follows:

**Table 2:** Confusion matrix.

Classification		Reference	
		CS-	CS+
Prediction	CS	TP	FP
	CS+	FN	TN

- TP (true positive): classification instances that the model identifies as positive and are indeed positive. classification instances that the model identifies as positive and are indeed positive.
- TN (true negative): classification instances that the model identifies as negative and are indeed negative.
- FP (false positive): classification instances that the model incorrectly identifies as positive but are actually negative.
- FN (false negative): classification instances that the model incorrectly identifies as negative but are actually positive (Table 2).

CS+: Cushing syndrome positive,

CS-: Cushing syndrome negative,

N = TP + FN + FP + TN

$$\begin{array}{l} Accuracy\;=\frac{TP+TN}{N} \end{array}$$ (3)

$$\begin{array}{l} Sensitivity=\;\frac{TP}{TP+FN} \end{array}$$ (4)

$$\begin{array}{l} Specificity=\frac{TN}{FP+TN} \end{array}$$ (5)

$$\begin{array}{l} Positive\;Predictive\;Value\;=\frac{TP}{TP+FP} \end{array}$$ (6)

$$\begin{array}{l} Negative\;Predictive\;Value\;=\;\frac{TN}{TN+FN} \end{array}$$ (7)

$$\begin{array}{l} Detection\;Rate\;=\;\frac{TP}{N} \end{array}$$ (8)

$$\begin{array}{l} Prevalence\;=\;\frac{TP+FN}{N} \end{array}$$ (9)

$$\begin{array}{l} Detection\;Rate\;=\;\frac{TP}{N} \end{array}$$ (10)

$$\begin{array}{l} Detection\;Prevelence\;=\;\frac{TP+FP}{N} \end{array}$$ (11)

$$\begin{array}{l} Balanced\;Accuracy\;=\;\frac{sensitivity+specificity}{2} \end{array}$$ (12)

$$\begin{array}{l} Kappa\;=\frac{Acc-EA}{1-EA} \end{array}$$ (13)

## 3. Results

### 3.1. Correlation between data characteristics

**Figure 1.**
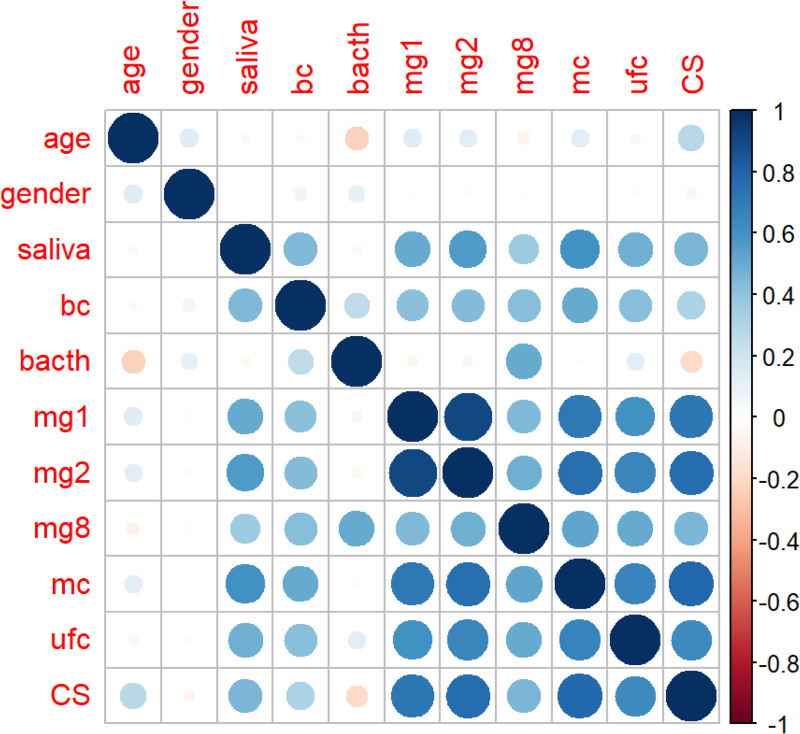
Correlation graph saliva:salivary cortisol, mg1 = 1 mg DST, mg2 = 2 mg DST, mg8 = 8 mg DST, mc = midnight cortisol, ufc = urinary free cortisol, CS = Cushing Syndrome (Since the first letters in a computer program cannot start with a number, “mg” was added before the numbers.)

A correlation graph illustrates the relationships between features, with positive relationships depicted in shades of blue and negative relationships in shades of red. The intensity of the color indicates the strength of the relationship between 2 features. Upon examining the correlation graph, several noteworthy observations emerge (Fig. 1).

Features exhibiting a positive correlation of over 70% with CS include 2 mg (78.8%), 1 mg (76.9%), and mc (72.1%). Additionally, 8 mg (45%) and saliva (45.4%) display positive correlations close to 50%.

Conversely, gender traits exhibit a correlation very close to zero with CS. This lack of correlation suggests that gender is not a significant independent trait and was consequently removed from the dataset.

### 3.2. GLM results

**Figure 2.**
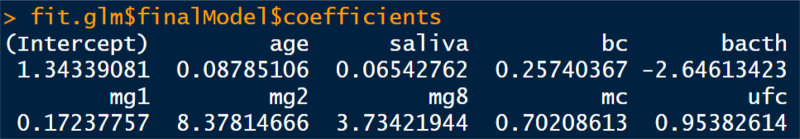
GLM coefficients: saliva = salivary cortisol, bc = Bazal Cortisol, bacth = Bazal ACTH, mg1 = 1 mg DST, mg2 = 2 mg DST, mg8 = 8 mg DST, mc = midnight cortisol, ufc = urinary free cortisol (Since the first letters in a computer program cannot start with a number, “mg” was added before the numbers.). DST = dexamethasone suppression test, GLM = Generalized Linear Models.

The coefficients of the model obtained after running the GLM algorithm in the RStudio environment are shown in Figure 2.^[45]^

Considering these coefficients, the mathematical expression given in formula (14) is obtained:

$$\begin{array}{l} \begin{array}{l} logit\left(\;p\right)≅1,343+0,088\log\left(age\right)+0,065\log\left(saliva\right)\;\\ \;\;\;\;\;\;\;\;\;\;\;\;\;\;\;\;\;\;+0,257\log\left(bc\right)-2,646\log\left(bacth\right)+0,172\log\left(mg1\right)\;\\ \;\;\;\;\;\;\;\;\;\;\;\;\;\;\;\;\;\;+8,378\log\left(mg2\right)+3,734\log\left(mg8\right)+0,702\log\left(mc\right)\;\\ \;\;\;\;\;\;\;\;\;\;\;\;\;\;\;\;\;\;+0,954\log\left(ufc\right)\; \end{array} \end{array}$$ (14)

This mathematical expression represents the relationship between CS and other variables. The coefficient of each variable represents the effect of that variable on CS. For example, since the coefficient of age is 0.088, the CS value is expected to increase by approximately 0.088 with increasing age. Variables with positive coefficients (age, saliva, bc, 1 mg, 2 mg, 8 mg, mc, ufc) increase the CS value, while the variable with a negative coefficient (bacth) decreases the CS value. The magnitude of the coefficients indicates the strength of the relationship. For instance, since the coefficient of the 2 mg variable is 8.378, its effect on CS is greater than that of the other variables.

### 3.3. Evaluation of the model

**Figure 3.**
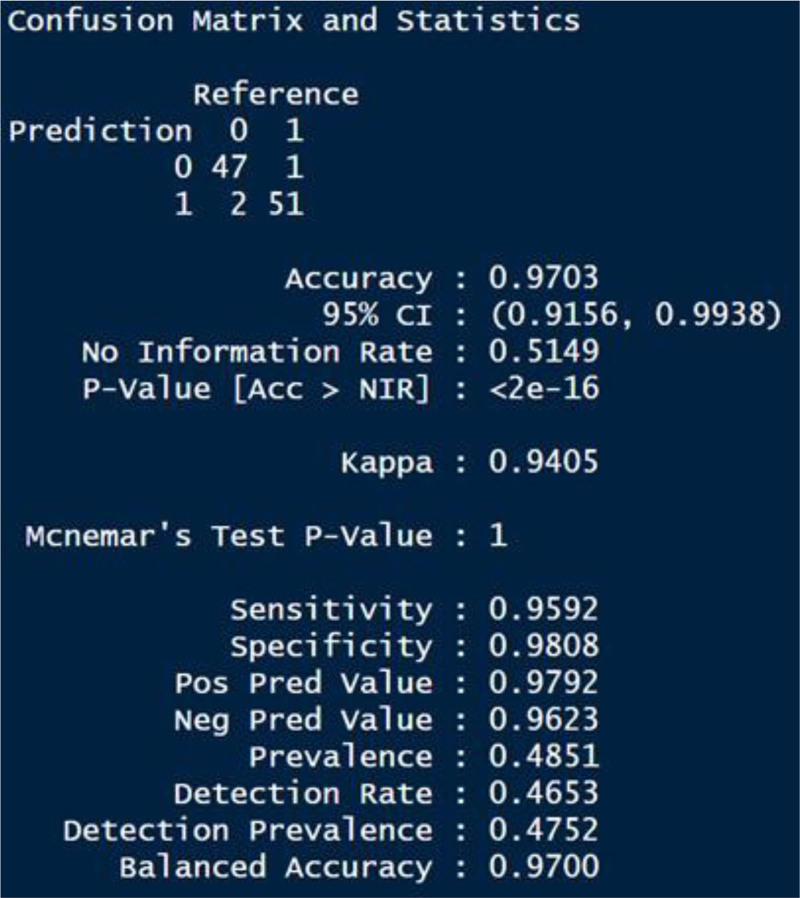
Test results of GLM model. GLM = Generalized Linear Models.

The significance of the model is assessed through the *P*-value. A *P*-value below .05 indicates significance, with lower values (closer to 0) indicating greater significance.^[46]^ As shown in Figure 3, our GLM model is statistically significant with a *P*-value of < 2.10^-16^, which is very close to zero. Once the model is deemed significant, metrics are calculated based on the Confusion Matrix values. The results obtained using formula (3–13) are shown in Figure 3.

When the Confusion Matrix is analyzed, 2 out of the 49 patients who are actually healthy (FN) are misclassified as patients. Similarly, 1 out of 52 patients (FP) was misclassified as healthy. Accordingly, the overall classification accuracy of the model is 97.03%.

### 3.4. Transferring the model to a mobile application

The mobile application for the obtained linear model was developed using App Inventor, an online design platform created by MIT.^[47]^ The mathematical model derived using GLM was implemented within the App Inventor design interface. After completing the necessary checks, an apk file was generated. This APK file was then transferred to a mobile device, installed, and executed.

**Figure 4.**
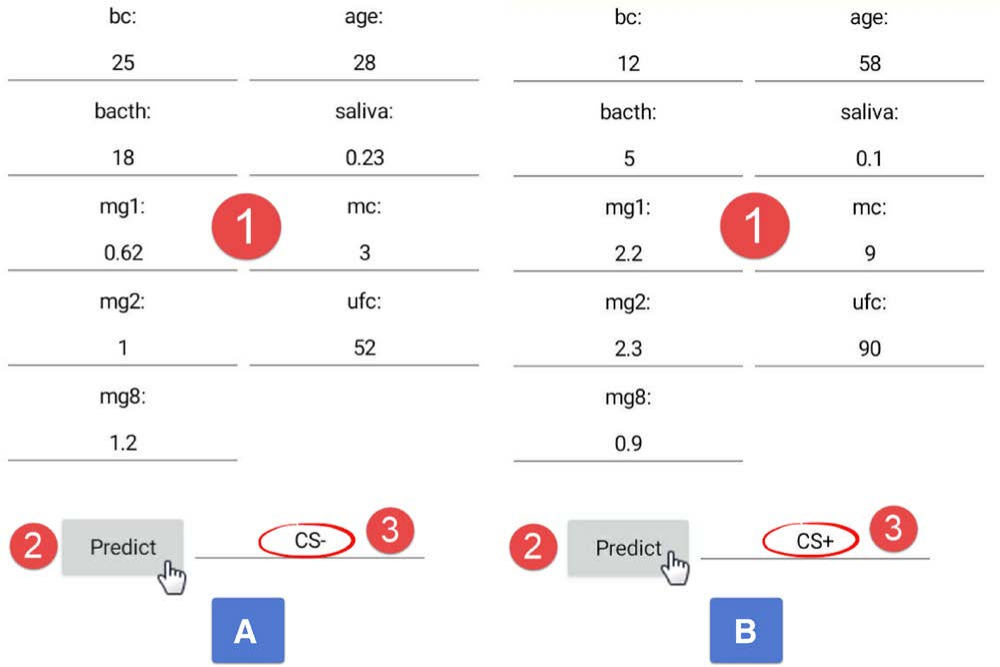
Image of the mobile application interface bc = Bazal Cortisol, bacth = Bazal ACTH, saliva = salivary cortisol, mg1 = 1 mg DST, mg2 = 2 mg DST, mg8 = 8 mg DST, mc = midnight cortisol, ufc = urinary free cortisol (Since the first letters in a computer program cannot start with a number, “mg” was added before the numbers.). DST = dexamethasone suppression test.

The visual interface that opens after running the mobile software is shown in Figure 4. In this interface, data for 9 separate parameters were entered for both an individual with CS (A) and a healthy individual (B) into the sections marked with number 1 in Figure 4. To perform the prediction, the “Predict” button marked with number 2 was pressed to execute the calculation.

In the result obtained for image A, it was revealed that the patient is CS-, as indicated by number 3. Similarly, in image B, the patient was predicted to be CS+, as indicated by number 3 (Fig. 4).

## 4. Discussion

In this study, a ML algorithm was developed using simple biochemical tests to diagnose CS and achieved an accuracy rate of over 97.3%. The results demonstrate that ML algorithms based on simple clinical tests can effectively diagnose CS. Furthermore, this algorithm has been made accessible through a user-friendly interface to all clinicians. The mathematical model, developed using GLM, was transferred as a mathematical expression in the App Inventor design interface, and an APK file was generated. The apk file was transferred to a mobile device and executed, resulting in a functional application that assists clinicians in diagnosing CS with a high accuracy rate.

Accurate diagnosis of CS is crucial for ensuring effective treatment for patients.^[20–22]^ Our study presents an innovative approach that leverages the advanced predictive capabilities of ML using basic biochemical tests for diagnosing CS. Additionally, the mobile application allows clinicians to quickly diagnose CS by inputting data on their phones, particularly useful in outpatient clinic conditions.

ML offers consistent analysis by processing large datasets without being constrained by rigid rules, unlike traditional methods that are more sensitive to user influence. The accuracy rates of biochemical tests used in the diagnosing CS were also examined. Features with more than 70% positive correlation with CS include 2 mg DST (78.8%), 1 mg DST (76.9%), and mc (72.1%), respectively. Additionally, 8 mg DST (45%) and saliva (45.4%) showed positive correlations close to 50%. In contrast, gender characteristics had a near-zero correlation with CS. The most valuable biochemical test was found to be the 2 mg DST.

In conclusion, ML significantly contributes to endocrinology by providing better and more accurate predictions in the diagnosis of CS. Previous studies have shown that ML outperforms conventional methods in predictive power, such as predicting surgical outcomes in patients with pituitary adenoma, postoperative remission in acromegaly, and resistance to somatostatin receptor ligands in acromegaly treatment.^[48–53]^ These studies consistently show that ML outperforms traditional methods in terms of predictive power. However, there are only a few studies in the existing literature that deal specifically with the application of ML in the context of CS. For example, Isci et al^[8]^ used ML algorithms to differentiate between CD, adrenal CS, and subclinical CS; however, their study did not include patients with EAS. In another study, Lyu et al^[54]^ used ML algorithms for the differential diagnosis of 311 patients with ACTH-dependent CS. They used the random forest method and achieved a correct diagnosis with a sensitivity of 95% and a specificity of 71.4%. However, these studies included parameters such as body mass index and disease duration, but did not include crucial biochemical parameters like the 1 mg DST, and MRI features like adenoma density. Demir AN et al^[55]^ used ML algorithms considering various factors including age, gender, serum potassium levels, screening, confirmatory and differential diagnostic tests for hypercortisolemia, along with sella MRI data, achieving an 86% accuracy rate in determining the etiology of ACTH-dependent CS.

Although factors such as biochemical tests, age, gender, and imaging modalities each have limitations in diagnosing CS accurately, ML combines all these features to perform comprehensive analysis and predictions. This balances the limitations of ML and its relatively limited utility in differential diagnosis. ML’s powerful data processing capabilities provide a valuable and noninvasive tool in contrast to the current medical approach, which faces challenges in effectively integrating these features for accurate decisions.

The interpretation of medical tests to diagnose CS and classify its subtypes is time-consuming and limited by physicians’ capacity and clinical experience to integrate numerous and complex pieces of information. Results from studies conducted in other hospitals and medical centers may vary due to factors such as laboratory errors, patient-induced errors, differences between groups, age and gender. Despite these challenges, we have demonstrated that an ML-based decision support system can be helpfull.

Class imbalance in the dataset is known to reduce the predictive performance of a model.^[56]^ Various methods are available to alleviate this problem such as subsampling and oversampling techniques. Since the dataset size is relatively small, subsampling the majority classes results in information loss. Oversampling is likely to bias the accuracy as new data samples are created from a few old samples and cannot introduce significant changes to the dataset. Therefore, we tackle this problem by using ML algorithms that can inherently handle imbalanced data classification through class weighting in the learning process. This ML approach outperforms expert human judgment in clinical performance evaluation.

One limitation of this study is a relatively small number of EC patients, which is reflective of clinical practice. Large-scale studies are needed to determine the robustness and reproducibility of the model, as the decision-making success of AI depends on the sample size and data amount. Another limitation is that the algorithm we developed should include all the necessary variables for effective clinical use.

However, the fact that all selected variables are easily accessible and inexpensive measurements that can be applied in outpatient clinic conditions will facilitate their use. Approximately 20% of ACTH-dependent cases are EC, while 80% are PC (i.e., Cushing Disease). Since both PC and EC are ACTH-dependent, some of the samples estimated as PC in the original dataset may actually be EC. Therefore, further studies by physicians are needed to make a correct diagnosis. Bilateral inferior petrosal sinus sampling (BIPSS), imaging findings, and the rate of suppression of cortisol levels after an 8 mg DST are generally informative in differentiating EC from PC. In our study, there were fewer data on BIPSS tests, imaging methods, and the 8 mg DST. As we collect more data samples for EC, the models can be easily updated to differentiate EC.

Despite these limitations, the ML model offers numerous important advantages. This research represents one of the initial successful implementations of ML in accurately predicting CS diagnosis. The model can serve as a valuable decision support tool for clinicians, especially when used in integrated with a mobile application, thereby simplifying the diagnosis of CS in situations where direct diagnosis is challenging. Our approach is adaptable to new data and will evolve with the accumulation of new samples. Once trained, the prediction models require minimal computational resources and the features are derived from routinely conducted tests in hospitals. It can also serve as a general framework, allowing the integration of data from various hospitals and medical centers. These models can help screen a large proportion of negative cases in the early stages of clinical diagnosis, prognosis, and treatment.

## 5. Conclusion

This study aimed to develop an effective method for predicting the diagnosis of CS and examining factors affecting its diagnosis, including age, gender, and biochemical tests. Using the GLM classification algorithm, researchers achieved a 97.03% accuracy in predicting the CS diagnosis. To accomplish this, they leveraged the power of AI to develop a ML algorithm that integrates clinical, biochemical and radiological data. After comprehensive testing, the algorithm demonstrated high performance in predicting the diagnosis of CS in both training and test datasets. The data obtained through this algorithm can be used to create a mobile application serving as a clinical decision support tool, promising more successful decision-making in the future. Additionally, this algorithm has been made accessible to all clinicians through a user-friendly interface. Although this classification model has achieved significant success, it is necessary to analyze and compare the results with other classification algorithms.

In this study, a lightweight and interpretable predictive model was developed using GLM to estimate cushing syndrome and this model was successfully implemented as a mobile decision support application using MIT App Inventor. The choice of GLM was motivated by its simplicity, transparency, and compatibility with low-resource mobile environments, making the solution suitable for real-time use by healthcare workers or caregivers in practical settings.

While GLM served as a valuable baseline in this context, future work will focus on exploring more advanced modeling approaches to improve prediction performance and flexibility. In particular, fuzzy logic-based models, such as fuzzy regression, hierarchical fuzzy systems, and TSK models, may offer significant advantages in handling uncertainty and non-linearity in medical data. These methods will be considered for integration in future versions of the application, especially if the deployment environment allows more computational capacity.

Moreover, deep learning techniques (such as attention mechanisms and transformer-based architectures) have demonstrated remarkable success in modeling complex and high-dimensional datasets. These approaches could be particularly beneficial in future studies involving large-scale patient data or temporal sequences, such as continuous monitoring of vital signs. Although these models currently exceed the computational limits of MIT App Inventor, future implementations using more flexible platforms (e.g., TensorFlow Lite or Flutter with on-device inference) may enable their integration into mobile health applications.

In summary, this work presents a foundational step toward accessible, mobile-based clinical decision support systems, with future directions aiming to incorporate advanced AI methodologies while maintaining usability and real-time responsiveness.

## 6. Limitations

Our study has several important limitations that must be acknowledged: the model was developed using data from a single medical center, which may limit its generalizability. For instance, cortisol measurements in our center were obtained using chemiluminescence immunoassays, while other centers may use tandem mass spectrometry. Differences in testing methods across centers could affect the model’s predictive performance. The dataset is relatively small for developing robust machine learning models. In particular, the limited number of patients with ectopic Cushing syndrome and endogenous Cushing may influence the accuracy and reliability of predictions. Larger, more diverse datasets are needed in future studies to enhance differential diagnosis. We did not exclude patients with cyclical CS from our dataset. Since their clinical variables are often unstable, it is unclear whether the model performs accurately for this subgroup. The model was developed with a relatively small number of EC patients, which may affect its robustness and reproducibility. Large-scale studies are necessary to validate its clinical utility. Important diagnostic tools such as BIPSS and imaging results were not incorporated into the machine learning model. Including these tools could improve diagnostic accuracy. While downsampling the majority class can lead to information loss, oversampling the minority class might introduce bias and reduce prediction accuracy. The algorithm must include all necessary clinical variables to ensure usability by physicians in real-world settings. The success of AI in medical decision-making relies heavily on sample size and data quality. More comprehensive studies using different approaches are essential for developing reliable diagnostic models.

## Author contributions

**Conceptualization:** Mustafa Aydemir, Mustafa Çakir, Okan Oral.

**Data curation:** Mustafa Aydemir, Mustafa Çakir.

**Formal analysis:** Mustafa Aydemir, Mustafa Çakir, Okan Oral, Mesut Yilmaz.

**Investigation:** Mustafa Aydemir, Mustafa Çakir, Okan Oral, Mesut Yilmaz.

**Methodology:** Mustafa Çakir, Okan Oral, Mesut Yilmaz.

**Project administration:** Mustafa Aydemir, Mustafa Çakir, Okan Oral, Mesut Yilmaz.

**Resources:** Mesut Yilmaz.

**Software:** Mustafa Çakir, Okan Oral.

**Supervision:** Mustafa Çakir, Okan Oral, Mesut Yilmaz.

**Validation:** Mustafa Aydemir, Mustafa Çakir, Okan Oral, Mesut Yilmaz.

**Visualization:** Mustafa Çakir, Okan Oral, Mesut Yilmaz.

**Writing – original draft:** Mustafa Aydemir.

**Writing – review & editing:** Mustafa Aydemir.
